# Exploring convergent validity of a multidimensional language screening-tool translated for use with two-year-olds in a migration context in Austria

**DOI:** 10.3389/fped.2026.1821578

**Published:** 2026-09-24

**Authors:** Daniel Holzinger, Laura Habermaier, Dajie Zhang, Christoph Weber

**Affiliations:** 1 Institute of Neurology of Senses and Language, Hospital of St. John of God, Linz, Austria; 2 Clinical Research Institute for Developmental Medicine, Johannes Kepler University Linz, Linz, Austria; 3 Institute of Linguistics, University of Graz, Graz, Austria; 4 Department of Child and Adolescent Psychiatry, Psychosomatics and Psychotherapy, Heidelberg University, University Hospital Heidelberg and German Center for Mental Health (DZPG), Heidelberg, Germany; 5 Department of Neurology, interdisciplinary Developmental Neuroscience (iDN), Medical University of Graz, Graz, Austria; 6 Child and Adolescent Psychiatry and Psychotherapy, University Medical Center Göttingen, German Center for Child and Adolescent Health (DZKJ), Göttingen, Germany; 7 Department for Inclusive Education, University of Education Upper Austria, Linz, Austria

**Keywords:** language screening, late language emergence, late talker, migration context, multilingualism, toddler, two-year-olds

## Abstract

**Introduction:**

Implementations of the SPES-2 language screening in primary pediatric care demonstrated good predictive validity and feasibility with monolingual German-speaking two-year-olds. This study examines the construct validity of translated versions of the instrument in children growing up with first languages other than German in a migration context.

**Methods:**

SPES-2 includes a parent-reported questionnaire and a direct assessment of word comprehension by the pediatrician. The instrument was translated into the seven most common minority languages in Austria and implemented across a range of primary care practices. For analyses, data for the four most common languages (Serbian-Bosnian-Croatian (SBC), Turkish, Albanian, Romanian; total *n* = 1,232) were compared with a monolingual German-speaking reference group (*n* = 2,758). Wordbank data were used as a reference for language development in the respective home countries.

**Results:**

Correlations between outcomes from the translated parent-reported SPES-2 word lists and other language assessments were statistically significant and of moderate to high effect size, demonstrating convergent validity. Using German cut-offs, word list results for SBC, Albanian and Romanian were slightly lower than those in German, and substantially lower in the Turkish sample, that could only be partially explained by differences in parental education. Comparisons of Wordbank data for the SPES-2 wordlist in German with Croatian and Turkish showed almost identical results for German and Croatian but lower results for Turkish, indicating a possibly linguistically inadequate composition of the Turkish word list. Comparisons between the findings for total vocabulary in our study samples and Wordbank data demonstrated slightly higher results for German and Croatian, but lower results for the Turkish migrant group.

**Discussion:**

Brief reports by parents of migrant origin on their two-year-old children's total vocabulary (and other language aspects) within regular pediatric well-baby check-ups seem to sufficiently reflect child language development. Comparative findings for vocabulary development by children with Turkish as their first language in a migration context demonstrate the need for linguistic adaptations of the screening tool. The SBC translation of SPES-2 does not appear to require cultural adaptations. For all translations, predictive validity for later language disorders needs to be examined.

## 1 Introduction

Almost 28% of the Austrian population has a migration background (1st or 2nd generation) (1). Approximately three in ten primary school children (30.9%) have a first language other than German. The most common non-German first languages in schools are Turkish and Serbian-Bosnian-Croatian (SBC), spoken by 6% and 6.9% of the children, respectively (2). The majority of families with a migration background use their heritage language and some German at home. Most children are largely monolingual in early childhood and acquire German after enrolment in preschools from about age three years. A recent study in Germany showed particularly high interest within the Turkish community in passing on their heritage language (approximately 80%) (3).

Children with language difficulties are at increased risk for longer-term problems, such as social isolation, mental health problems (4–6), school problems (7, 8) and unemployment (9, 10). Consequently, developmental language difficulties are both a personal and a public health concern.

The majority of children growing up multilingually acquire their dominant language within the typical benchmarks of monolingual language development and can be classified as typical learners (11). Nevertheless, early access to two or more languages is not a guarantee of successful multilingual development. On the contrary, children from bilingual immigrant families who grow up with a heritage language that differs from the majority language often reach school age with insufficient proficiency in both languages (12), which may cascade into long-term problems mentioned above.

Late language emergence (LLE) is usually defined by an expressive vocabulary of fewer than 50 words, and/or the lack of word combinations at 24 months, with an absence of other primary impairments that would explain the language delay (13, 14). Expressive language alone may be affected, or the delay may include receptive language, in which case the risk of persistent language disorder is even greater (15). A growing body of evidence demonstrates the effectiveness of parent-mediated early intervention in enhancing language development in children with LLE (16–21).

Given the significant impact that early identification and subsequent intervention can have on children's functional outcomes, the American Academy of Pediatrics has recommended the implementation of systematic developmental surveillance (22). However, even for monolingual children, universal screening for language delay has not been officially recommended to date. Two systematic reviews by the US Preventative Services Task Force (23, 24) reported insufficient evidence regarding the feasibility and impact of currently available language screening measures on child language development. Several studies have demonstrated the validity and reliability of early language screening—mostly for monolingual children—using parent reports (25–27), including the MacArthur-Bates Communicative Development Inventories (MCDI) (28) with adaptations in over 60 languages, such as Turkish (TIGE) (29) and German (FRAKIS) (30). Other less comprehensive parent-reported language assessment tools have been developed and validated for German. ELFRA (Elternfragebogen; Parent Questionnaire) (31) is a 260-item word list based on the MCDI and Language Development Survey (LDS) (14), standardized for use with 2-year-old children (32). Additionally, the short parent-reported screening tool SBE 2 KT (Sprachbeurteilung durch Eltern Kurztest; short language assessment by parents) has been validated (33) for German-speaking children. Recently, Holzinger et al. (34) demonstrated the validity and feasibility of a two-stage screening procedure for use with two-year-olds (SPES-2; Sprachentwicklungsscreening; Screening of language development). SPES-2 includes, in stage 1, a parental report of expressive vocabulary, word combinations and concerns about language. Stage 2 is a brief direct assessment of word comprehension conducted by the pediatrician. SPES-2 demonstrated predictive validity in identifying significant language delay at age three years. To date, however, no validated screening measure is available for children growing up in a German-speaking country with a dominant heritage language other than German.

### 1.1 Explaining late language emergence

Risk factors for LLE can be related to the child (e.g., male sex and birth order) and to the family (23, 35, 36). For both monolingual (34) and bilingual (37) children, parental concerns about language development are strongly associated with LLE. Although socioeconomic status (SES) has a significant impact on language development, studies on language acquisition in migration contexts have shown that SES does not fully explain differences in language development between monolingual and multilingual children (38). Some studies have not even found a significant effect of higher parental education in bilingual samples, neither on child vocabulary (39, 40) nor on grammar (41) acquisition.

One key environmental factor in children's early language skills is parental linguistic input, in which quality (i.e., variety of vocabulary, syntactic complexity, use of facilitative language techniques) and quantity (i.e., number of words/sounds/utterances) are usually strongly correlated (42, 43). For two-year-old children with a migration background, the frequency of activities involving interaction with an adult, including shared book reading, correlated significantly with expressive vocabulary in the majority language at 30 months of age (40). However, there appears to be considerable cultural diversity in the importance of direct parental knowledge transfer to children and shared book reading (44, 45). Scheele et al. (46), for example, showed that, compared to monolingual Dutch children, bilingual children in the Netherlands with Moroccan and Turkish first languages received significantly less language input in both their first (L1) and second language (L2) via book reading and a range of oral language interactions, such as personal conversations and oral storytelling. For children growing up with multiple languages, input time is generally divided among several language systems (37). In addition, the richness of input can be reduced due to the limited number of native speakers in the child's environment (38, 47, 48). Bohnacker et al. (49) showed that parental language choice strongly affects children's expressive vocabulary. Children growing up in Sweden with German and Turkish L1 had significantly higher vocabulary scores in their first languages when their parents used only the heritage language with their child and with each other. Parents who speak a minority language themselves often do not have native-level proficiency in the majority language and may feel uncomfortable speaking it to their children. Both the quality and amount of input in the majority language may therefore be limited (50).

An additional influencing factor associated with migration and living in a foreign country is parental stress. Some evidence suggests that postnatal maternal stress may negatively impact a child's language development (51). The systematic review by Fair et al. (52) identified stressors experienced by migrant women in relation to pregnancy, childbirth and maternity care in European countries; these included navigating a foreign health system, language barriers, cultural insensitivity and prejudice, financial constraints and previous traumatic experiences related to migration.

Summing up, LLE refers to a delay in the acquisition of early language milestones (particularly for vocabulary and word combinations) that can have manifold correlates in the child and the environment. Environmental effects on language acquisition of children in migration contexts relate particularly to language input that can be reduced or divided between several languages. LLE is associated with an increased risk of persisting language delay, however, at the age of two, it should not generally be considered as pathological.

Given the abundance of factors influencing early language development in a migration context, identifying children at increased risk of persistent language delay is a major challenge. As a consequence of language input divided among different languages, screenings must take two (or more) input languages into account. Two measures have been proposed to quantify bilingual children's combined vocabulary: Conceptual vocabulary measures the number of concepts rather than words, with concepts represented in both languages counted only once (37). However, Core et al. (53) contended that acquiring two phonological forms for a given concept represents an autonomous linguistic proficiency, and therefore advocated assessing total vocabulary*,* including enumerating individual lexical entries in each language. The majority of studies support measuring total vocabulary, particularly for young children (up to the age of about two years), resulting in mean expressive vocabulary scores and average growth rates similar to those in monolingual development (53–55).

This study sought to achieve four goals: First, we assessed the construct validity of the translated versions of the SPES-2 language screening during regular well-child check-ups for children with a migration background growing up in Austria. The original SPES-2 was translated into the seven most commonly spoken non-German languages in Austria at the time of data collection—Turkish, Serbian-Bosnian-Croatian (SBC), Albanian, Romanian, Russian, Arabic and Spanish. Convergent validity of the translated versions was examined by assessing correlations of the respective SPES-2 expressive vocabulary score and other SPES-2 measures considered potential indicators of LLE.

Second, we investigated variance in expressive vocabulary by migration background. Specifically, we first examined differences between the monolingual German sample and the samples with an L1 other than German, and then described differences within the non-German language groups.

Third, we investigated whether potential differences in expressive vocabulary between the language groups could be explained by sociodemographic child and family variables (i.e., child age and sex, firstborn status, parental education).

Fourth, to explore the linguistic and cultural fairness of the SPES-2 word list, we examined vocabulary differences across three language groups (German, Turkish and Croatian) with available MCDI Wordbank data (56) at the single-word level.

## 2 Methods and procedures

### 2.1 Measures

The SPES-2 language-screening for monolingual German-speaking two-year-olds was developed for use in pediatric primary care during regular well-child check-ups. The screening tool (34) consists of a parent-reported questionnaire, originally available and validated in German, and translated into the seven most commonly spoken non-German languages in Austria. The questionnaire covers (i) sociodemographic information of the family (highest level of parental education on a six-level scale ranging from “no degree” to “university degree” and the birth order of the child), (ii) information about the primary language used in the family and the child's best language. To assess the child's language skills, parents complete (iii) a 37-item word list of the child's expressive vocabulary (36 nouns and 1 verb drawn from various semantic fields, such as body parts, food, clothes, toys, furniture, vehicles and everyday life). Parents assess the child's production of each vocabulary item in both languages (i.e., child's first language and German), (iv) report the child's use of two-word combinations, (v) indicate parental concerns about language development (typical, slightly delayed, severely delayed) and (vi) estimate their child's language development (no—some—major delay).

Since word comprehension is considered to contribute to predicting language disorder (15, 34), a brief word comprehension subtest from a standardized German-language test [SETK-2 (57);] was selected for direct pediatric assessment. The pediatrician presents single spoken words to the child (nine items in total; Cronbach's alpha = 0.69) referring to household items, food, animals and body parts, and the child is asked to identify the corresponding picture from four options. The SPES-2 screening yields a three-stage result indicating low, moderate, or high risk. For children with lows risk rescreening at the age of three years (SPES-3) is recommended. For parents of children with moderate risk, preventive parent counseling or parent training by speech-language professionals (promoting responsive interaction and facilitative language techniques within everyday family routines and dialogic book reading) is offered, whereas high-risk children are referred for multiprofessional developmental assessment.

### 2.2 Procedures

In Austria, the universally accessible health system provides preventive health care for all children from birth up to the age of five years. Data collection for the German reference sample took place between fall 2011 and spring 2014, whereas SPES-2 data collection for children with non-German L1 spanned a longer period of time (from 2011 to 2020) to attain a sufficient sample size. In 2009, the Pediatric Association of Upper Austria invited all primary care pediatric offices to participate in a longitudinal language-screening project. Thirty pediatricians (about 50% of all primary care pediatricians in Upper Austria) from across Upper Austria agreed to screen all children aged two and three years for language delay as part of their regular well-baby checkups. Pediatricians were trained in administering the final screening procedures, scoring, and communicating results to parents, and were provided with the screening materials.

In stage 1 of the SPES-2 screening, parents completed the short parent questionnaire in the waiting room while awaiting the regular two-year well-child check-up. An assistant then collected the completed forms. In stage 2, pediatricians directly assessed the child's receptive vocabulary. If German was not the child's dominant language, a simple written translation of the target words (e.g., show me rabbit, apple, knife) was provided to a caregiver fluent in the child's native language and, on behalf of the pediatrician, they asked the child to identify the corresponding pictures from the given options. Parents were instructed to use the provided word list rather than baby-talk expressions (such as yum-yum to indicate food or moo for a cow) and/or supporting gestures for the respective concepts. Before the actual screening, one training item was administered by the parent as instructed by the pediatrician. When parents used words deviating from the word list, parents were asked for clarification, and dialectal variations were accepted. After each assessment, completed parental screening forms were sent to the Hospital of St. John of God in Linz.

### 2.3 Participants

A total of *n* = 2,109 screening forms for children growing up in a multilingual context were collected in the pediatric offices, of which 1,305 met the inclusion criteria. Inclusion criteria were age at the time of testing between 22 and 26 months, according to the time window recommended for the well-baby check-up around age 2, and the time window for the validation of the German SPES-2 screening. Additional inclusion criteria were the availability of the child's sex, and a clear affiliation to a non-German language group. Parents were asked whether their child grew up with more than one language, and if so, which language they considered to be the child's best language for assignment to a language group. Parents of two-year-old children can generally provide reliable information about their child's relative proficiency across languages. However, it must be noted, that a combination with standardized language measures in both languages is more informative, particularly when one language is used primarily outside the home. In the Austrian context it is still rare for a child to be in daycare outside the family at the age of 2 years.

Participants from smaller language groups (Russian *n* = 30, Arabic *n* = 34, Spanish *n* = 9) were excluded from further analyses because of insufficient sample sizes. The remaining sample of 1,232 multilingual children belonged to the following language groups: Turkish (*n* = 424), SBC (*n* = 490), Albanian (*n* = 179) and Romanian (*n* = 139). The sample sizes mirror the distribution of the four most common language groups among children in Upper Austria with a migration background from non-German speaking countries (25.9% with SBC background, 14.9% with Turkish background, 9.1% from Albanian-speaking countries (i.e., Albania and Kosovo), and 7.9% with Romanian background (58) ^1^.

Additionally, a reference group of *n* = 2,758 monolingual German-speaking children from a previous validation study of the monolingual SPES-2 screening (34) was included. The final study sample included 3,990 participants across the multilingual and reference groups (see Figure 1).

**Figure 1:**
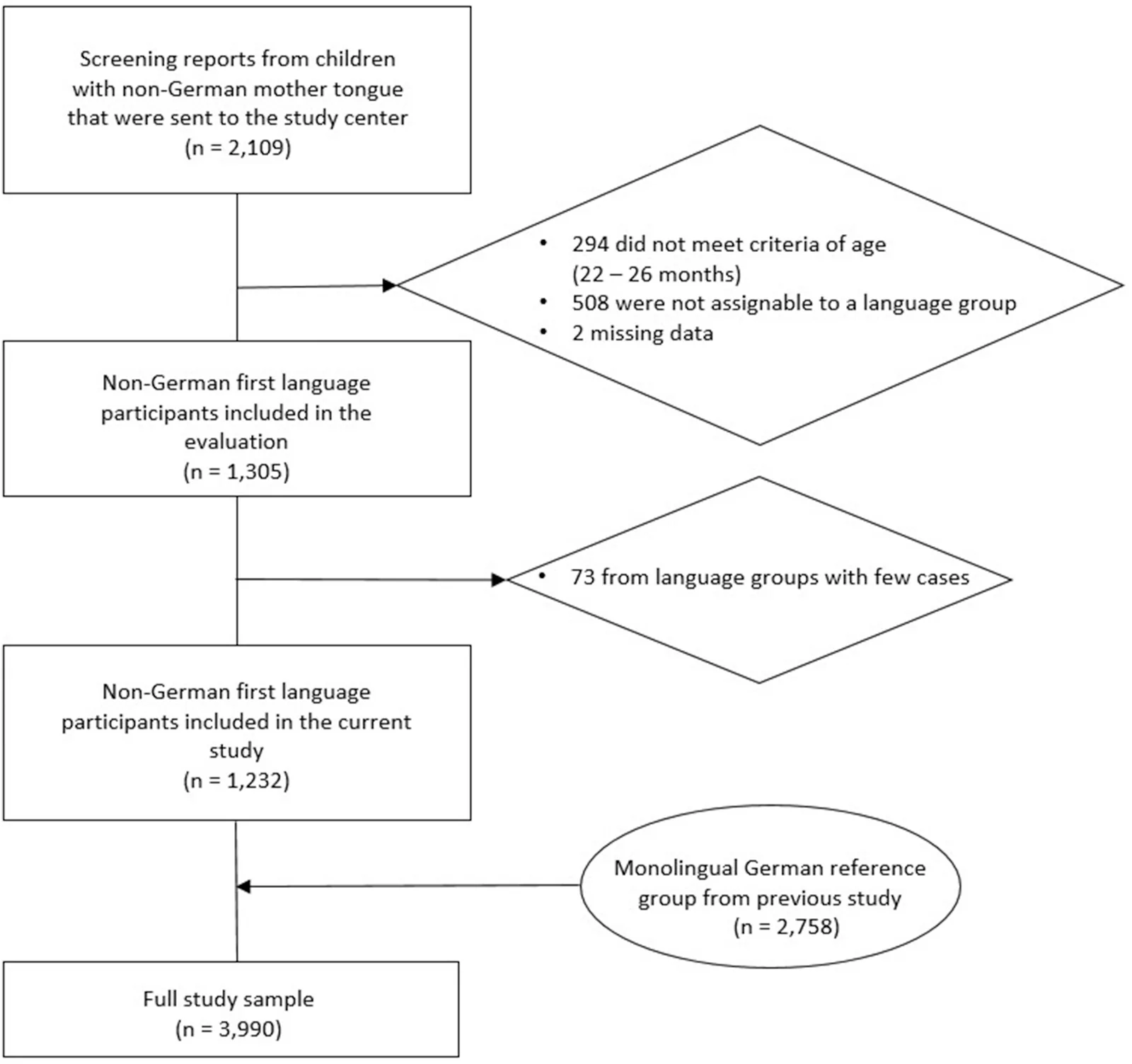
Participant recruitment.

### 2.4 Statistical analysis

Although data were complete for the expressive vocabulary list, language group, child gender, and child age, missingness ranged from 1.5% (firstborn status) to 29.8% (pediatrician-assessed word comprehension), indicating limitations of direct language assessment within the constraints of primary pediatric care and challenges of including parents in the direct assessment. As indicated by Little's Missing Completely at Random (MCAR) Test (59) implemented in SPSS, variable means significantly differed between missing data patterns (*χ*^2^(231) = 503.05, *p* < .001), which suggested a Missing at Random (MAR) mechanism. Among others, parents of children with missing word comprehension scores reported lower levels of education and lower expressive vocabulary. To adequately address the missing data under the MAR mechanism, we applied multiple imputation (MI; (60)). MI is a state-of-the-art procedure that outperforms older methods (e.g., listwise deletion) and yields (approximately) unbiased estimates under MAR even with substantial missingness (60). In our data, the highest missing rate was 29.8%, a level where MI is well suited. In detail, we used Blimp 3 software (61) to generate 20 multiply imputed datasets using a fully conditional specification approach. All variables included in this study were used in the imputation model.

To analyze multiply imputed data, we used the structural equation modeling (SEM) framework of Mplus 8 (62). The command TYPE = IMPUTATION pools the results of the 20 imputed datasets according to Rubin (1987 (63); see also (60)).

To assess the validity of the expressive vocabulary list, we estimated its correlation with other language-assessment variables (the child's use of two-word combinations: yes = 0, no = 1; parental concerns about language development: no concerns = 0, some or major concerns = 1; parental estimation of their child's language development: typical = 0, slightly or severely delayed = 1; pediatrician-assessed word comprehension ranging from 0 to 9) within each language group. To investigate language differences in criterion validity, we computed correlation differences (effect size q; (64)) supplemented by confidence intervals as proposed by Zou (65) and implemented in the cocor R Package (66).

Second, we used a multigroup SEM-approach to evaluate differences between language groups in expressive vocabulary and the language assessment variables. Specifically, we compared means for continuous variables (corresponding to ANOVAs) and proportions for categorical variables (corresponding to cross-tabulations) between language groups by testing whether all group differences differ from 0. This was done using the MODEL TEST command, which applies a Wald-*χ*^2^ test.

Third, we used multiple regression to investigate whether language group differences in expressive vocabulary can be explained by group differences in parental education (see Table 1). To this end, we first entered sociodemographic background variables (age, gender, and firstborn status; see Table 1) to control for language-group differences in variables that are known to be associated with expressive vocabulary (67). We then entered the language group to estimate differences between groups, controlling for the background variables. We subsequently entered parental education. Any changes in the expressive vocabulary differences between language groups would then be due to differences in education. Wald-*χ*^2^ tests were used to investigate whether the step wise included predictors are associated with an increase in variance explained (R^2^).

**Table 1:** Sociodemographic sample characteristics .

Sociodemographic variables	German (*n* = 2,758)	Turkish (*n* = 424)	SBC (*n* = 490)	Albanian (*n* = 179)	Romanian (*n* = 139)	Total (*n* = 3,990)
Age in months M (SD)	23.90 (.90)	23.93 (.91)	24.01 (.80)	23.96 (.82)	24.06 (.81)	23.93 (.88)
Male sex %	52.0	58.5	48.6	54.2	51.8	52.4
Firstborn %	50.5	31.1	38.0	32.4	45.3	45.7
Highest parental education %						
Compulsory lower secondary education	2.8	24.9	3.9	20.5	10.3	6.2
Upper secondary education (vocational schools)	29.3	27.6	34.1	9.4	6.6	28.1
Postsecondary education (ISCED 4)	16.8	4.7	21.7	43.3	25.0	17.6
Upper secondary education with university entrance qualification	21.0	34.3	26.7	12.9	27.9	23.0
Tertiary education	30.1	8.5	13.6	14.0	30.1	25.1

Finally, we applied ANOVAs in SPSS 29 to examine group differences in vocabulary using open-source raw data from the MCDI Wordbank (56).

## 3 Results

### 3.1 Participant characteristics

Table 1 provides an overview of the sample characteristics. The language groups differed in highest parental education, with the largest proportion of parents who completed only compulsory lower secondary education in the Turkish group (*χ*^2^(16) = 704.26, *p* < .001; Cramer V = .209). Comparing parental education with reference values^2^ from the Austrian educational standard tests in primary school in 2018^3^, the German-speaking sample can be regarded as representative in terms of parental education (*χ*^2^(3) = 10.07, *p* > .05), with similar proportions of parents with tertiary education (30.1% in our sample vs. 29.3%). Similarly, the Turkish group in our sample had a proportion of tertiary-educated parents comparable to Turkish parents in the Austrian sample (8.5% and 7.7%, respectively). However, Turkish parents with higher levels of education (e.g., upper secondary education with university entrance qualification) were overrepresented in our sample (34.3% vs. 12.0% in the Austrian sample), while parents with compulsory lower secondary education were underrepresented (24.9% vs. 41.6% in the Austrian sample; *χ*^2^(3) = 198.85, *p* < .001). For the SBC group, parents with tertiary education were also overrepresented in our sample (13.6% vs. 7.8% in the Austrian sample; *χ*^2^(3) = 34.15, *p* < .001).

Significant differences between language groups were also found for other sociodemographic variables. For instance, the proportion of boys (58.5%) was higher among Turkish-speaking children than among the other groups (*χ*^2^(4) = 84.948, *p* < .001, Cramer V = .148). Moreover, there were more firstborn children in the German-speaking group than in the bilingual groups (*χ*^2^(4) = 9.599, *p* < .05, Cramer V = .049).

### 3.2 Convergent validity of translations of the expressive vocabulary list

Convergent validity of the translations of the German SPES-2 word list into Turkish, SBC, Romanian and Albanian was examined by analyzing correlations of expressive vocabulary with other measures that have been highly predictive of language delay, including parental report on language delay (no, minor, major), parental concerns about language development (no, minor, major), parental report on the occurrence of spontaneous two-word utterances (no, yes), and results of the stage 2 pediatric screening for delays in receptive vocabulary (raw score). Furthermore, these values were compared to those obtained for the German word list.

The correlations between expressive vocabulary and the other language assessment variables are reported in Table 2. Overall, the correlations for the languages other than German are all statistically significant (*p* < .01) and of moderate to large effect sizes (median *r* = |.309|; r_min_ = |.263|; r_max_ = |.539|). Correlations between parental reports of expressive vocabulary and the occurrence of two-word utterances in the child's spontaneous language production were found to be strong for all languages and of moderate or large effect sizes (*r* > |−.405|). This is similar to the value obtained from the German reference group. However, for parent-reported delayed language development and parental concerns, correlations with expressive vocabulary are higher within the German language group than within the other groups. According to the test for correlation differences, these differences in correlation are statistically significant for the Turkish, SBC and Albanian groups. The greatest difference was found for parent-reported delayed language development for the SBC group (effect size for correlation differences *q* = −.257; 95%-CI [−.282, −.120]). Supplementary regression analyses showed that the associations between expressive vocabulary and (a) parent-rated language delay and (b) parental concern are moderated by parental education (b_language delay × education_ = −1.234, *p* < .001; b_concerns × education_ = −1.364, *p* < .01), with weaker associations at lower levels of parental education. That is, the weaker correlations are associated with the larger proportion of parents with lower levels of education among parents with a migration background,

**Table 2:** Correlations of expressive vocabulary with other language assessment variables by language groups.

Languages	Lack of two-word utterances^a^	Delayed language development^b^	Parental concerns^b^	Word comprehension^b^
German	−.585***	−.566***	−.421***	.336***
Turkish	−.532***	−.387***	−.286***	.316***
SBC	−.539***	−.367***	−.285***	.263***
Albanian	−.405***	−.376***	−.288***	.337**
Romanian	−.487***	−.435***	−.344***	.302**
Correlation differences				
German vs.	q [95%-CI]	q [95%-CI]	q [95%-CI]	q [95%-CI]
Turkish	−.077 [−.129,.018]	−.233 [−.267, −.097]	−.155 [−.230, −.044]	.022 [−.070,.114]
SBC	−.067 [−.116,.019]	−.257 [−.282, −.120]	−.156 [−.225, −.051]	.080 [−.014,.164]
Albanian	−.240 [−.313, −.062]	−.246 [−.323, −.068]	−.153 [−.277, −.0002]	−.001 [−.129,.140]
Romanian	−.138 [−.238,.022]	−.176 [−.278, −.003]	−.090 [−.236,.065]	.038 [−.114,.197]

q = effect size for correlation differences; 95%-CI = confidence interval for correlation differences as suggested by Zou (65).

a point-biserial correlation.

b Pearson correlation.

** *p* < .01.

*** *p* < .001.

As delays in expressive vocabulary are related to language comprehension difficulties only in a proportion of children with LLE (68), a weaker correlation may be expected between word comprehension and expressive language than for the other measures involved. This was reflected in our results (Table 2). Nonetheless, correlations for word comprehension and expressive language ranged from *r* = .263 (SBC) to *r* = .337 (Albanian). These values were all statistically significant and were comparable to the German sample (*r* = .336).

In sum, the convergent validities of translated versions of SPES-2 in four languages (i.e., Turkish, SBC, Albanian and Romanian) resemble the validity of the original German version.

### 3.3 Language results by language groups

Parents from the German language group reported that their children used about 29 out of the 37 words (M = 28.91, SD = 10.67), whereas children from the Turkish group produced about nine words fewer (M = 19.58, SD = 13.93) (Table 3). The expressive vocabulary scores for the other language groups ranged from M = 25.85 (SD = 14.98, SBC) to M = 23.63 (SD = 13.68, Romanian). The group differences were highly significant (Wald-*χ*^2^(4) = 48.603, *p* < .001). Based on pairwise group comparisons, the differences between the German group and each of the other language groups, as well as the Turkish group and each of the other language groups, were highly significant (*p* < .001). Effect sizes (Cohen's d) ranged from small (German vs. SBC: d = 0.256, *p* < .001) to large (German vs. Turkish: d = 0.783, *p* < .001). Differences between SBC, Romanian and Albanian groups were not significant. Further analyses showed that in all multilingual groups the number of expressive German words was low, and was the lowest in the Turkish group.

**Table 3:** Language group differences in expressive vocabulary and other language assessment variables.

Language assessments	German (*n* = 2,758)	Turkish (*n* = 424)	SBC (*n* = 490)	Albanian (*n* = 179)	Romanian (*n* = 139)
Expressive Total Vocabulary M (SD)	28.91 (10.67)	19.58 (13.93)	25.85 (14.98)	25.02 (14.05)	23.63 (13.68)
Expressive Vocabulary German M (SD)	28.91 (10.67)	2.72 (6.49)	4.79 (8.40)	4.95 (8.55)	4.78 (9.15)
Delayed language development % (parent report)	14.2	20.4	15.6	15.8	21.3
Parental Concerns %	9.4	19.6	10.4	15.9	15.1
Lack of two-word utterances % (parent report)	12.9	37.0	28.0	25.1	26.6
Word comprehension M (SD) (direct assessment)	7.73 (1.55)	6.17 (2.01)	6.87 (1.80)	6.74 (1.79)	6.85 (1.89)

Similarly, there were significant group differences in word comprehension (Wald-*χ*^2^(4) = 245.701, *p* < .001), with the highest scores in the German group (M = 7.73, SD = 1.55) and the lowest scores in the Turkish group (M = 6.17, SD = 2.01). Again, a group comparison revealed that the means for monolingual German-speaking children were significantly higher than the means for the other language groups. The Turkish group scored significantly below the other non-German groups (at least *p* < .01). Differences between the German group and the other groups were of moderate (German vs. SBC: d = 0.519, *p* < .001) to large size (German vs. Turkish: d = 0.945, *p* < .001).

With respect to the parent-reported potential signs for LLE, we found significant group differences in delayed language development (Wald-*χ*^2^(4) = 14.137, *p* < .01), parental concerns (Wald-*χ*^2^(4) = 40.588, *p* < .001), and lack of two-word utterances (Wald-*χ*^2^(4) = 176.466, *p* < .001). All the potential indicators of LLE were least frequently reported by the monolingual German group, and most frequently by the Turkish language group (Table 3). Detailed analyses show a low number of German words included in the total vocabulary of the non-German language groups (see Table 3, row Expressive Vocabulary German), most pronounced in children growing up with Turkish L1 (13.9% (=2.72/19.58*100) for the Turkish, 18.5% for SBC, 19.1% for Albanian, and 20.2% for Romanian L1).

### 3.4 Explaining expressive vocabulary differences by sociodemographic characteristics

Results of the multiple regression model examining whether group differences in expressive vocabulary can be explained by differences in sociodemographic variables are reported in Table 4. In step 1, child-related demographic variables (i.e., age, firstborn status, gender), which indeed differed between language groups, had significant effects on expressive vocabulary, but accounted for only 4.0% of the total variance. Older age at the time of assessment, being firstborn and being female had a positive impact on early expressive vocabulary. Entering the language group in step 2 (reference grou*p* = German) increased R^2^ by.056 (*p* < .001), indicating that age, gender, firstborn status, and language group together explained 9.6% of the variance in expressive vocabulary. As indicated by the regression coefficients B, the differences between the German and the other groups were similar to the bivariate differences reported above (see Table 3). Finally, entering parental education in step 3 increased the explained variance significantly by 1.2% (*p* < .001) to a total of 10.8%. Notably, parents with compulsory lower secondary education reported about five words fewer (*B* = −4.743, *p* < .001) than parents with postsecondary education. The impact of language groups remained statistically significant at *p* < .001 and the respective regression coefficient B changed only slightly. In particular, from Step 2 to Step 3, the difference between German- and Turkish-speaking children decreased from *B* = −8.937 (*p* < .001; standardized difference d = −0.750) to *B* = −7.659 (*p* < .001; *d* = −0.642). Thus, a difference of about one word (−8.937—(−7.659) = −1.278) can be explained by differences in the education of German- and Turkish-speaking parents. Likewise, from the initial *B* = −3.710 (*p* < .001; d = −0.311) difference between the Albanian and the German groups, about one word could be explained by parental education differences (step 3, *B* = −2.751, *p* < .01; *d* = −0.231). Finally, differences in expressive vocabulary between the German and the SBC and the Romanian groups changed little with the parental educational levels^4^.

**Table 4:** Hierarchical regression results for total expressive vocabulary.

	B	95% CI for B		SE B	d^a^	R^2^	*Δ*R^2^
		LL	UL				
Step 1							
Constant	27.184	26.810	27.558	0.191		.040	.040***
Age	1.605***	1.180	2.029	0.216			
Firstborn	1.972***	1.211	2.733	0.388			
Gender (male)	−3.470***	−4.219	−2.721	0.382			
Step 2							
Constant	28.886	28.448	29.324	0.223		.096	.056***
Age	1.685***	1.272	2.097	0.210			
Firstborn	1.194**	0.448	1.940	0.381			
Gender (male)	−3.297***	−4.025	−2.569	0.371			
Non-German language group (reference = German)							
Turkish	−8.937***	−10.142	−7.731	0.615	−0.750		
SBC	−3.212***	−4.341	−2.083	0.576	−0.269		
Albanian	−3.710***	−5.484	−1.937	0.905	−0.311		
Romanian	−5.502***	−7.496	−3.508	1.017	−0.462		
Step 3							
Constant	28.661	27.736	29.586	0.472		.108	.012***
Age	1.646***	1.236	2.055	0.209			
Firstborn	1.058**	0.316	1.801	0.379			
Gender (male)	−3.315***	−4.038	−2.592	0.369			
Non-German language group (reference = German)							
Turkish	−7.659***	−8.922	−6.395	0.645	−0.642		
SBC	−2.919***	−4.048	−1.789	0.576	−0.245		
Albanian	−2.751**	−4.558	−0.944	0.922	−0.231		
Romanian	−5.302***	−7.294	−3.310	1.016	−0.445		
Parental Education (reference = Postsecondary education (ISCED 4))							
Compulsory lower secondary education	−4.743***	−6.543	−2.943	0.918			
Upper secondary education (vocational schools)	−0.556	−1.685	0.574	0.576			
Upper secondary education with university entrance qualification	0.357	−0.825	1.539	0.603			
Tertiary education	1.500*	0.345	2.655	0.589			

CI, confidence interval; LL, lower limit; UL, upper limit. ^a^ d, standardized difference, calculated as B/(pooled standard deviation).

a Significance for *Δ*R^2^ was calculated using a Wald-*χ*^2^ Test, which tests that all entered predictors have null effects.

** *p* < .01.

* *p* < 0.05.

*** *p* < .001.

### 3.5 Exploring differences in expressive vocabulary results between language groups based on reference data of monolingual children

To better understand whether the differences in expressive vocabulary after controlling for education mirror specifics of language acquisition observed in the respective countries of origin or are due to an insensitive selection of words, we used data from the MacArthur-Bates Communicative Development Inventory Wordbank (56) for the available languages German (30), Turkish (29) and Croatian (69). Sample sizes for the target age range are *n* = 480 (German), *n* = 609 (Turkish), and *n* = 139 (Croatian). Notably, available data refer to adapted word lists that consist of different numbers of words for each language group. Whereas the word list contains 588 items for German-speaking children, the archive data for Turkish-speaking and Croatian-speaking children include 711 and 717 items, respectively. Further, the words included differ between language groups, and we found exact matches across all three languages only for 247 words. To account for differences between word lists and to investigate possible effects of the 37 selected words—for which not all words were available across all three language groups (35 words for German, 37 words for Turkish, 36 words for Croatian)—we conducted a set of comparisons. The results are shown in Table 5.

**Table 5:** CDI wordbank data of expressive vocabulary in two-year-old monolingual children as a comparison of SPES-2 data in the current study.

		German (*n* = 480)	Turkish (*n* = 609)	Croatian (*n* = 139)	Group differences	Effect size^a^	
						G vs. T	G vs. SBC
A	Wordbank Total	239.39 (157.41)	267.86 (204.22)	275.30 (149.20)	–		
B	Wordbank Total % of available items	40.71 (26.77)	37.67 (29.23)	38.40 (20.81)	F_Welch_(2, 437.327) = 1.665, *p* = .190; *η*^2^ = .003	−0.129	−0.094
C	Wordbank (247 shared items)	116.86 (70.82)	104.09 (74.83)	115.52 (56.12)	F_Welch_(2, 429.262) = 4.683, *p* = .010; η^2^ = .008	−0.179	−0.019
D	Wordbank (37 SPES items)	26.82 (10.55)	23.05 (11.32)	26.65 (7.45)	F_Welch_(2, 456.434) = 19.253, *p* < .001; η^2^ = .030	−0.354	−0.016
E	Wordbank (37) % available items	76.63 (30.15)	62.30 (30.59)	74.04 (20.70)	F_Welch_(2, 454.729) = 33.490, *p* = .010; η^2^ = .052	−0.486	−0.088

a effect size (d) estimated as mean difference divided by the pooled standard deviation.

First (see row A), on average two-year-olds growing up monolingually with German produced 234.65 (of 588) words (SD = 153.65), Turkish children 262.55 (of 711) words (SD = 204.22) and Croatian children 267.68 (of 717) words (SD = 145.35). Given the differences in the number of words assessed, we calculated the percentage of words produced to allow meaningful comparison (see row B). German-speaking children produced 40.71% (SD = 26.77) of the test words, Turkish-speaking children 37.67% (SD = 29.23) and Croatian-speaking children 38.40% (SD = 20.81). These differences are not significant (see group-differences column), which indicates high similarities in early vocabulary acquisition for children growing up monolingually with the respective languages in their countries of origin. Second (see row C), to further facilitate comparison, we compared the means for the 247 words that were included in the word lists of all three languages. ANOVA revealed significant group differences (see group- differences column), with the highest means among German-speaking children (M = 116.86, SD = 70.82) and the lowest for Turkish-speaking children (M = 104.09, SD = 74.83). After all, the difference between German and Turkish-speaking children was small (d = -.179). However, the differences became more pronounced when comparing the number of spoken items of the list of 37 words of the SPES screening (see rows D and E). German-speaking children produced on average 76.63% of the 35 available SPES screening words, which is more than Turkish-speaking children (62.30% of the 37 available SPES screening words). To conclude, some differences between German-speaking and Turkish-speaking children may be due to the selection of the 37 words following the original German SPES screening, as indicated by larger differences in the Wordbank 37 word list (d = −.486) than in the 247 shared word list (d = −.179). The small but significant differences in the 247 shared word list may reflect unmeasured confounders in the Wordbank data (e.g., parental education). Finally, comparing the word bank data with the results reported in the preceding part (see Table 3) indicates that especially Turkish-speaking children with migration background scored about three words lower than the monolingual Turkish-speaking population represented by the Wordbank (M = 19.58 vs. M = 23.05; d = −.278, *p* < .001). This difference is even more remarkable given that the total vocabulary (including Turkish and German words for the SPES screening) was compared with the monolingual Turkish vocabulary (Wordbank). Notably, vocabulary scores of the German-speaking and the Croatian-speaking Wordbank samples did not differ significantly from the total vocabulary scores of the SPES-samples^5^.

## 4 Discussion

This study investigated the suitability of using translations of the German SPES-2 language screening within primary pediatric care to identify LLE in two-year-olds growing up with a family language other than German in Austria. The SPES-2 was translated and examined here for four different languages: Turkish, Serbian-Bosnian-Croatian, Albanian and Romanian. The original SPES-2 has shown high predictive accuracy and practicability within regular well-child checkups for monolingual children acquiring German (34). For Aim 1 of the study, the convergent validities of the translated word lists (37 words) were confirmed for all four languages. In particular, the correlation between expressive vocabulary and the other potential signs for LLE was statistically significant and of moderate to large effect size. For all four languages, the observed correlation pattern (i.e., between expressive vocabulary and other potential signs for LLE) resembled that of the original SPES-2 for monolingual German-speaking children. Therefore, the translated expressive vocabulary short checklist, counting spoken words both in the heritage language and in German (total vocabulary) alongside translations of the other components of SPES-2, appears to be a valid method for early language screening for two-year-old bilingual children growing up in Austria.

Another aim (Aim 2) of the study was to compare expressive vocabulary ranges across language groups to examine the suitability of German cut-offs for other languages. The scores of the total expressive vocabulary (including both heritage language and German) of the four groups of bilingual children were found to be significantly lower than those of monolingual German-speaking children. In particular, the score was significantly lower in Turkish-speaking children than in children growing up with SBC, Albanian or Romanian as their L1. Similar patterns were found for word comprehension, with German-speaking children scoring the highest and Turkish-speaking children the lowest. Regression analyses to explain the differences in expressive vocabulary (Aim 3) showed that, after controlling for child age, firstborn status and gender (explaining 4.0% of the variance) and language group (increasing the explained variance to 9.6%), parental educational level increased the explained variance only marginally by 1.2%. Child sociodemographic variables and parental education thus failed to adequately account for the variations observed in expressive vocabulary between monolingual and multilingual children. A partial explanation of the differences between the language groups might be related to the specific input conditions of language acquisition in a migration context such as the amount of child directed parental language (42, 43), the number of available native speakers or the frequency of activities involving parent-child interaction (including dialogic bookreading) (40).

To explore language-specific differences in the acquisition of early vocabulary (Aim 4) that might explain differences in vocabulary ranges measured by the short SPES-2 word lists, we first used the comprehensive MCDI word lists that have been linguistically and culturally adapted for German, Croatian and Turkish, and compared the proportions of words from these lists produced by two-year-old children from the respective language groups. We did not find significant differences between the language groups indicating comparable vocabulary size of two-year-olds across the languages. However, a reduction of the MCDI word list to the words (*n* = 247) shared by all three language groups showed statistically significant but small differences between the language groups, with the highest scores for German and the lowest for Turkish-speaking children, indicating cultural differences in the order of vocabulary acquisition. Similarly, Wordbank data for the 37 SPES-2 items showed almost identical results for German and Croatian with a significantly lower vocabulary score in Turkish. This suggests that the 37 items may not sufficiently capture the typical expressive words of normally developing 2-year-old monolingual Turkish children. Differences in the conceptual makeup of early vocabulary between German and Turkish languages, such as a higher proportion of verbs, furniture and people categories in the latter (70), might explain the disadvantaging effect of using simple translations from German. Notably, the German SPES-2 word list includes only one verb reflecting the noun-bias of early vocabulary acquisition in German as compared to the Turkish verb-bias. Thus, a mere translation of the 37 items rather than an adequate adaptation reflecting language-specific early vocabulary acquisition may underestimate the actual expressive vocabulary of Turkish-learning children.

On the other hand, a comparison between the Turkish Wordbank data and the Austrian data for the 37-item wordlist showed significantly lower scores in Turkish children growing up in Austria as compared to the norm sample from Turkey. This discrepancy was not found in children with Croatian background. Before assuming a specific early disadvantage of children with Turkish origin growing up in Austria, explanations in addition to item-bias, such as dialect mismatch or parent-report differences need to be considered. Nevertheless, a relative delay of early language development as compared to other migrant groups is affirmed by the Turkish children's other language outcomes (see Table 2: parental concerns about language, parental report of a language delay, existence of word combinations, word comprehension scores collected through direct assessment) below those of the other migrant groups. Empirical studies in German-speaking countries have consistently shown that children of Turkish origin tend to underperform in language proficiency and educational attainment compared to other migrant groups (71–73). In the current study, these disparities could only be partially explained by lower parental SES of Turkish families with often limited formal education and working in low-skilled sectors (74). Similarly, Bohnacker, Lindgren & Öztekin (49) found small but non-significant effects of parental education on Turkish in Swedish-Turkish children in the lexical domain. For Austria, less than half of the differences in reading outcomes between children with and without a migrant background (73) (extracted from OECD data) could be explained by parental education. Nevertheless, parental education indirectly shapes the home learning environment including quality and quantity of language exposure in the family (75, 76). Family educational orientation and cultural values have also been described to affect language and education outcomes with recent studies indicating a shift towards higher educational aspirations in Turkish-origin families (77). Limited early contact with the majority language, demonstrated by the very low number of German words in the language samples of two-year olds with Turkish L1 might be another contributor to the low total vocabulary scores at age 2 (78). Exposure to the majority language (German) most often begins in preschools above the age of three years.

This study is of particular significance, as there is a paucity of studies that investigate delays in very early language acquisition at 24 months of age in a migration context. Furthermore, it includes the comparison of four different non-German language groups of considerable sample sizes with a large German normative sample. In addition to expressive vocabulary, supplementary data associated with elevated risks for late language emergence were collected for convergent validation.

It was not feasible in this study to collect information about familial language use, parental language input, parental German and heritage language proficiency, literacy practices, day care exposure, migration generation and migration stressors in families with a migration background, which must be considered a limitation. Due to a lack of valid MCDI data for Albanian and Romanian, the analysis of the influence of a specific language and culture on early productive vocabulary was restricted to German, Turkish, and SBC. In addition, comparability between Wordbank and SPES samples might be limited due to unmeasured confounders, e.g., different SES profiles and other factors related to different recruitment strategies.

## 5 Conclusion

Brief parental reports of expressive vocabulary aggregating spoken words in both the heritage and the majority languages during regular well-child checkups for young children with a migration background demonstrate fair to good convergent validity for language development screening at two years of age. Despite including words in L1 and L2 (total vocabulary) expressive-vocabulary scores of the SBC, Albanian and Romanian groups growing up in Austria were slightly below those of children with German L1, which might be an effect of the migration context and indicate the necessity for a slight adaptation of the cutoffs avoiding misclassification as LLE. Highly similar results for the number of SPES-2 items in the Croatian and German Wordbank norm groups appear to indicate cultural fairness of the wordlist translated into Croatian. However, a comparison of the Turkish vocabulary outcomes of children growing up in Turkey and in Austria indicates that a mere translation most likely disadvantages children of Turkish origin growing up in a migrant context. Therefore, on the one hand, an adaptation of the word list to the specific conceptual makeup of early vocabulary in Turkish is required, and an adaptation of cut-offs informed by Turkish Wordbank data should be considered. On the other hand, the low early-vocabulary scores among children of Turkish origin can only be partly explained by the composition of the word list or parental education. Even though additional unknown factors might limit comparability of Wordbank and SPES samples, higher rates of children with delays in early language learning that warrant family-centred intervention cannot be excluded. For the use of translated minority language versions of SPES-2 as pediatric screenings, their validity to predict further language development still needs to be established.

## Data Availability

The raw data supporting the conclusions of this article will be made available by the authors, without undue reservation.

## Footnotes

^1^We thank the Federal Institute for Quality Assurance of the Austrian School System (IQS) for providing data on the distribution of language groups based on the educational standards assessment in primary schools in 2018.

^2^Provided on a 4-point scale. For comparison, we combined upper and postsecondary education.

^3^We thank the IQS for providing data on parental education based on the educational standards assessment in primary schools in 2018.

^4^Notably, because the data-collection window differs between the German and bilingual groups, we conducted several sensitivity analyses. First, within each language group we tested whether expressive vocabulary scores varied over time using ANOVAs; time was binned into two-year intervals. For the SBC group we observed a trend toward lower scores in more recent periods (Welch’s F(4, 215.23) = 4.59, *p* = .002; η^2^ = .034). Second, we estimated a regression with time fixed effects and, separately, re-estimated the step-3 model using weights that align the bilingual groups’ time distribution with that of the German group. Both strategies imply comparisons between language groups at the assessment years observed for the German group. Additionally, we fit a regression including a group × time interaction to allow group-specific time trends. Consistent with the ANOVAs, all three specifications yielded smaller differences between the SBC and German groups (*b* = −1.316, *p* > .05 for the fixed-effects model; *b* = −0.427, *p* > .05 for the weighting approach; *b* = −0.507, p > .05 for the group × time model). However, with the available data we cannot determine whether these patterns reflect genuine time trends or other factors such as changes in sample recruitment.

^5^The mean for the German-speaking SPES sample was 28.91 (corresponding to 78.13% of the 37 words). The mean for the German-speaking Wordbank sample was 26.82 (corresponding to 76.63% of the available 35 words). The difference is not significant (t(3236) = 1.045, *p* > .05). The mean for the SBC-speaking SPES sample was 25.85 (corresponding to 69.13% of the 37 words). The mean for the Croatian-speaking Wordbank sample was 26.65 (corresponding to 74.04% of the 36 available words). Again, the difference was not significant (t(627) = 1.376, *p* > .05).
